# Cross-Talk Correction Method for Knee Kinematics in Gait Analysis Using Principal Component Analysis (PCA): A New Proposal

**DOI:** 10.1371/journal.pone.0102098

**Published:** 2014-07-08

**Authors:** Audrey Baudet, Claire Morisset, Philippe d'Athis, Jean-Francis Maillefert, Jean-Marie Casillas, Paul Ornetti, Davy Laroche

**Affiliations:** 1 Centre d'Investigation Clinique INSERM 1432, Plateforme d'Investigation Technologique, Dijon University Hospital, Dijon, France; 2 INSERM U1093, Dijon, France; 3 Medical Information department, Dijon University Hospital, Dijon, France; 4 University of Burgundy, Dijon, France; 5 Department of rheumatology, Dijon University Hospital, Dijon, France; 6 Department of physical medicine, Dijon University Hospital, Dijon, France; University of Toronto, Italy

## Abstract

**Background:**

In 3D gait analysis, the knee joint is usually described by the Eulerian way. It consists in breaking down the motion between the articulating bones of the knee into three rotations around three axes: flexion/extension, abduction/adduction and internal/external rotation. However, the definition of these axes is prone to error, such as the “cross-talk” effect, due to difficult positioning of anatomical landmarks. This paper proposes a correction method, principal component analysis (PCA), based on an objective kinematic criterion for standardization, in order to improve knee joint kinematic analysis.

**Methods:**

The method was applied to the 3D gait data of two different groups (twenty healthy subjects and four with knee osteoarthritis). Then, this method was evaluated with respect to three main criteria: (1) the deletion of knee joint angle cross-talk (2) the reduction of variance in the varus/valgus kinematic profile (3) the posture trial varus/valgus deformation matching the X-ray value for patients with knee osteoarthritis. The effect of the correction method was tested statistically on variabilities and cross-talk during gait.

**Results:**

Cross-talk was lower (p<0.05) after correction (the correlation between the flexion-extension and varus-valgus kinematic profiles being annihilated). Additionally, the variance in the kinematic profile for knee varus/valgus and knee flexion/extension was found to be lower and higher (p<0.05), respectively, after correction for both the left and right side. Moreover, after correction, the posture trial varus/valgus angles were much closer to x-ray grading.

**Conclusion:**

The results show that the PCA correction applied to the knee joint eliminates the cross-talk effect, and does not alter the radiological varus/valgus deformation for patients with knee osteoarthritis. These findings suggest that the proposed correction method produces new rotational axes that better fit true knee motion.

## Introduction

3D video-based biomechanical models are usually used to evaluate knee motion during gait. It has proved challenging to measure and then to represent this movement [1], [2]. 3D knee joint kinematics is commonly expressed using Eulerian or Cardanic description [3]. It requires two coordinate systems (CS), each one associated with an articulating bone of the knee (femur or tibia). Both are defined by positioning reflective markers on the skin on specific bony landmarks [4]–[8], according to a standard arrangement (marker set). The motion of the distal segment relative to that of the proximal segment, calculated from the coordinates of the markers, is usually reported as three sequential rotations (Euler angles) around three axes: flexion-extension, abduction-adduction and internal-external rotation [9].

Consequently, this technique has to rely on the palpation of external anatomical landmarks, which is an experimental problem [10]. Given the reality of experimental conditions with different examiners and a succession of subjects to examine, this is a serious limitation. Minor changes of marker placements modify the orientation of the coordinate systems and thereafter lead to significant errors in abduction/adduction and internal/external rotation angle curves [11]–[14]. This error is known as the kinematic “cross-talk” effect [15]–[18], which particularly affects the kinematics of joints that articulate principally around one major component, e.g. the knee joint [19].

This error leads to overestimation of the ab/adduction and int/external rotation angles [11]–[13], [20]–[22]. Numerous methods have been proposed to correct this error in the pre-trial phase [23]–[27] while few methods have investigated correction during the post-trial phase [28], [29]. Actually, Woltring proposed to rotate the initial CS until ab/adduction and int/internal rotation are zeroed at the time of maximum knee flexion during gait [29]. In turn, Rivest reduced between-subject variance by minimizing the quadratic variations in the knee abduction/adduction and internal/external rotation angles [28]. However, even though these methods appreciably reduce the cross-talk effect, they may delete existing mechanisms of the pathological knee. Thus, there is still no clear optimal ad hoc method of correcting knee joint motion for purposes of quantitative gait analysis.

The aim of this study is to propose a new method to correct the angles of knee rotation using principal component analysis (PCA) [30]. PCA is a classical statistical method that comes from the mechanical concept of “inertia”. It was first formulated thus: “finding lines and planes of closest fit to systems of points in space” [31]. Applied to the knee, it would provide a better representation of knee angles, by transforming the original data of correlated angles into new ones with uncorrelated components.

## Materials and Methods

### Subjects

Subjects were randomly selected among patients between 50 and 75 years old from two cohorts of healthy (HE) subjects and knee osteoarthritis (OA) patients. The first group of 20 healthy subjects had normal knee function and the second group (4 patients hereafter referred to as OA1 to OA4) had knee OA and symptomatic varus/valgus deformities. The study was conducted in compliance with the principles of Good Clinical Practice and the Declaration of Helsinki and all patients signed an informed consent form. The study protocol was approved by the local ethics committee (CPP Est I, Dijon, France) and was registered under ClinicalTrials.gov Identifier: NCT01884883.

### Data collection

**Figure 1:**
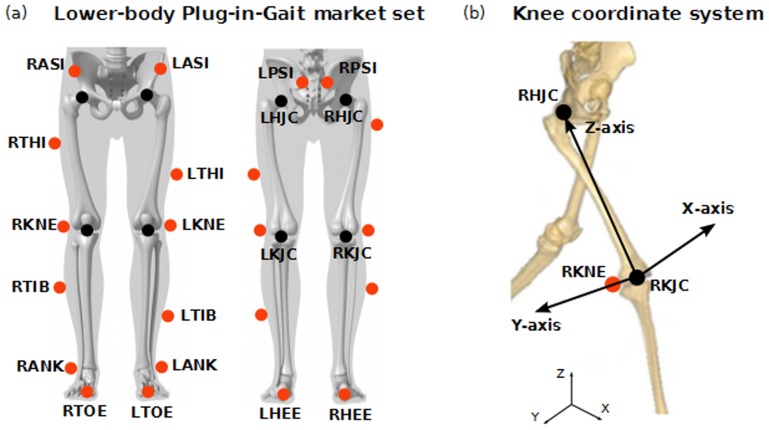
Plug-in-Gait marker set.

Kinematic data were captured using a three-dimensional (3D) Vicon motion analysis system (©VICON Motion Systems Ltd, UK) with eight cameras operating at a sampling rate of 100 Hz, mounted approximately 2.5 m high encircling the 6 m long×3 m wide×2 m tall capture volume. The marker set used for the study was the lower-body Plug-in-Gait provided by Vicon, based on the Newington-Helen Hayes gait model [12], [32] (Figure 1.a).

Particularly, the Vicon Plug-in-Gait applies a specific “chord function” in the calculation of the knee joint center (KJC). It estimates the hip joint center (HJC) using four markers on the pelvis and then uses the derived HJC, two other markers (thigh and knee markers) and determined offsets from the subject's measurements to estimate the KJC. The KJC is a point located half a knee width away from the knee marker placed on the lateral epicondyle of the femur. Then, the origin of the knee joint CS is taken as the KJC. The primary Z-axis is taken from the KJC to the hip joint center. The secondary Y-axis is taken parallel to the line from the KJC to the knee marker. For both left and right knees, the Y-axis is directed towards the left of the subject. The X-axis is hence directed forwards from the knee (Figure 1.b).

In each gait protocol, the test session began with a trial to capture static anatomical landmarks for calibration purposes. Then, instructions were given to the participants to walk in a comfortable manner, at a self-selected speed along a 10 m walking path. Each participant performed twenty gait trials. In addition, the four OA patients executed a thirty-second static posture trial in a radiological position in order to compare varus/valgus angles with the measured X-ray angle (see point 3 on 1.5 Statistical evaluation paragraph).

Anatomical varus/valgus angles were assessed on an antero-posterior weight-bearing knee X-ray with a size ratio of 1∶1. Knee valgus/varus misalignment was measured accurately in millimeters by an expert rheumatologist using a 0.1 mm graduated magnifying glass, laid directly over the radiograph. In this study, this measurement was considered the gold-standard for assessing valgus/varus misalignment.

### Data analysis

Three-dimensional knee joint angular kinematic data were obtained by tracking the trajectories of 14 mm spherical retro-reflective markers. These were mounted over the seven body segments of interest (pelvis, left and right thighs, left and right shanks, left and right foot; for detailed body landmarks see Figure 1).

Coordinate data were filtered using smoothing splines [33]. For missing markers on less than 10 frames, data were interpolated with cubic spline polynomials. Gait cycles containing gaps over 10 frames or more were automatically rejected from the analysis. Temporal events were defined using the position of the heel, toe and sacrum marker [34]. Each stride was time-normalized to 101 points representing equal intervals from 0% to 100% of the gait cycle.

### Correction procedure

- Principal Component Analysis

The main goal of PCA is to find a space as close as possible to the observed points [35]. If X is the matrix for n observations by p variables 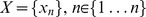, the q principal axes 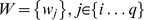 are orthonormal axes defining such a space. In physics, this means maximizing the inertia of a set of material points around these principal axes.

The vectors {*w_j_*} are given by the q dominant eigenvectors (i.e. those with the largest associated eigenvalues λ) of the covariance matrix of the set of observations:
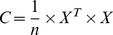
(1)The eigenvalues λ reflect the variance contribution (δ_j_) of the j-th principal axis as follows:
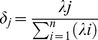
(2)The variables *x_n_* are then linearly transformed into uncorrelated variables for which the covariance matrix is diagonal with elements λ. Finally, it turns out that the q principal components of the observed vector *x_n_* are given by: 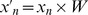.

- PCA calculations

The input data of the PCA analysis were the three angles of the knee joint, i.e. the Euler angles associated with the rotation between the articulating bones of the knee, over the 101 measurements of a gait cycle (*n* gait cycles for each subject). It was displayed as *n* (101×3) matrices for each subject:
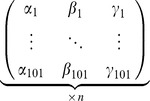
with α being the motion of flexion/extension, β of ab/adduction and γ of axial rotation.Then, once the matrix of covariance of this set of points has been calculated (see Equation 1), the eigenvalues and the components (a, b, c) of the eigenvectors may be determined in the observational basis:
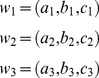
(3)This makes it possible to create the transfer matrix from the observational basis to the principal axes (Equation 4) and to calculate the principal components using the P matrix.
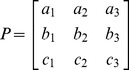
(4)Note that the principal axes and the transfer matrix have no physical sense but only a mathematical sense.

- Correction hypothesis

It is hypothesized that each principal component is a corrected anatomical angle.

Therefore, the eigenvectors must be sorted in order to match each principal component to an anatomical angle: the columns of the P matrix are switched depending on the values of the coefficients *a_i_*, *b_i_*, *c_i_* in such a way that the values of the diagonal are maximal.Finally, the corrected angles are determined using the “sorted” P matrix:



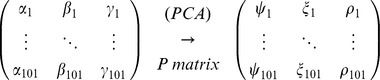
with *ψ* being the corrected flexion/extension angle, *ξ* the corrected ab/adduction angle and *ρ* the corrected axial rotation angle.

### Statistical evaluation

The method was evaluated on the kinematics of the two groups of subjects to comply with 3 requirements:

(1) The correction must remove cross-talk. In order to quantify the cross-talk, the relationship between the flexion-extension and varus-valgus kinematic profiles was assessed by the correlation between both profiles (r^2^), as reported by Schache et al [26].

(2) The variance in the varus/valgus kinematic profile must be minimal. The ab/adductor motion of the knee is physiologically limited, in asymptomatic condition, to approximately 7° due to the restrictions imposed by knee geometry and biomechanical properties [11], [18], [36]. Thus, an effective correction has to reduce the varus/valgus range of motion (ROM) resulting from cross-talk [23]. To check this, we computed the variance in its kinematic profile (δ^2^ab/ad). As a result of this reduction in varus-valgus ROM, the knee flexion-extension ROM has to be increased. To corroborate this, the variance in the flexion-extension kinematic profile (δ^2^f/e) was also calculated. Previous studies have used these criteria for a similar purpose [25], [28], [37], [38].

(3) In the case of symptomatic knee OA patients, it is essential for the varus/valgus deformities to remain after correction. The posture trial knee angles (see 1.2 Data collection paragraph) were thus corrected using the transfer matrix P (see 1.4.2 PCA calculations paragraph). We assumed that the varus/valgus angle obtained in the posture trial, after correction, would be close to the x-ray angle because of the similar position for both evaluations. Comparisons were made between the corrected posture angle and the anatomical angle assessed by x-ray.

### Statistical analysis

Parameters (variabilities δ^2^ and cross-talk r^2^) were averaged for all subjects and presented with the standard deviation (SD). Non-parametric tests for paired samples were used to detect whether the correction method had a significant effect: the Wilcoxon test for variabilities and the Friedman test for cross-talk. Comparisons were done for the left and right side separately. Differences were considered significant for p<0.05. Statistical analyses were performed using *Statistica 10.0*.

## Results

For every subject, 42±19 gait cycles were available, which was sufficient to obtain good reliability of knee joint angles and to overcome intrinsic variability [39].

**Figure 2:**
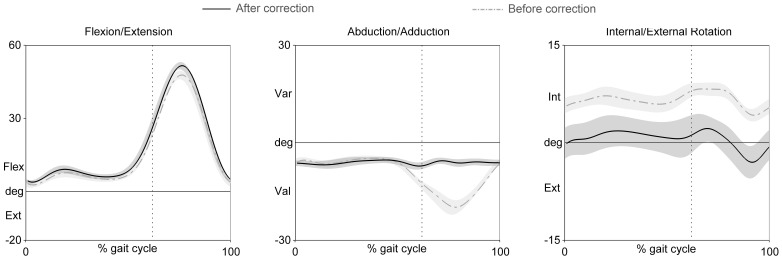
Mean and standard deviation of knee joint rotations during gait for a single typical subject before (grey dash-dotted line and light-grey area) and after correction (black solid line and grey area). Dotted vertical line indicates the mean toe off value for this subject.

Figure 2 shows the impact of the cross-talk correction on rotations of the knee. These results are consistent with previous studies [2,12,23.] They demonstrated that the more misaligned the defined CS, the more sensitive to change in flexion/extension the knee varus/valgus became. In contrast, knee axial rotation tended to be offset by approximately 5° but appeared to be less influenced by changes in knee flexion/extension.

The correction method eliminated the presence of knee joint angle cross-talk, as proved by mean r^2^ values close to 0 for the left and right side after correction. Cross-talk was significantly lower (p<0.0001) than before correction with mean r^2^ higher than 0.6 (table 1).

**Table 1:** Mean and S.D. of variability of the flexion/extension axis, the abduction/adduction axis and cross-talk (r^2^) between those axes, before (initial) and after (PCA-corrected) correction with PCA method.

Parameter	Initial		PCA-corrected	
	Mean	SD	Mean	SD
Variability δ^2^				
Flex/extension				
Right	16.47	1.82	17.54 *	1.45
Left	16.97	1.30	17.63 *	1.32
Ab/adduction				
Right	3.82	3.15	0.73 *	0.35
Left	3.36	2.07	0.93 *	0.50
Cross-talk (r^2^)				
Right	0.71	0.33	0 *	0
Left	0.60	0.39	0 *	0

*: significant difference (p<0.00625) between initial and PCA-corrected.

Moreover, the variances in the kinematic profiles for knee varus-valgus (δ^2^ab/ad) and knee flexion-extension (δ^2^f/e) were found to be significantly (p<0.05) lower and higher, respectively, after correction than before correction for both the left and right side (table 1).

**Figure 3:**
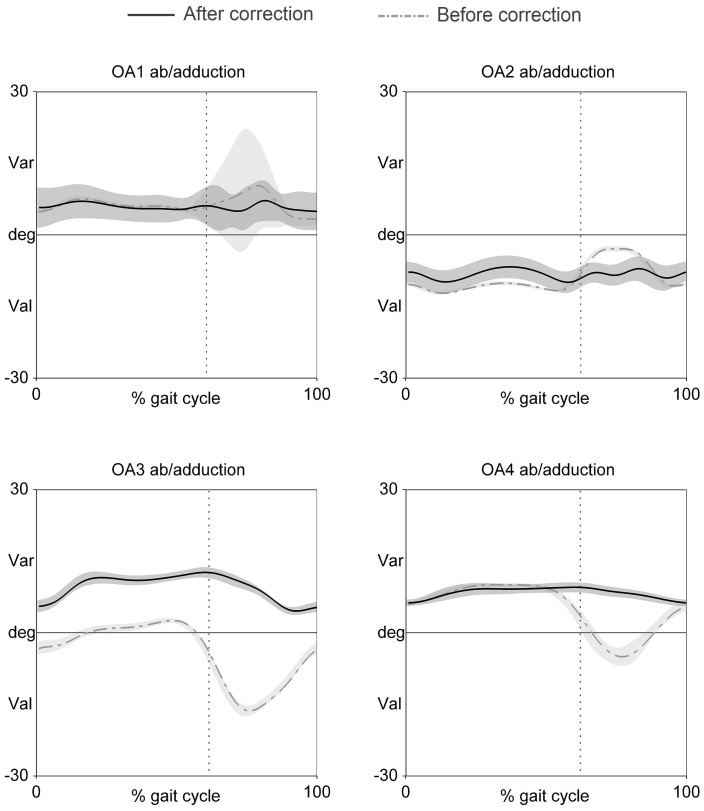
Averaged ab/adduction angle (mean and SD) during gait for each of the four OA patients before (grey dash-dotted line and light-grey area) and after correction (black solid line and grey area). Dotted vertical line indicates mean toe off.

To ensure that the PCA did not induce an offset of the abduction/adduction angles, we transferred (see methods) the reorientation of the knee CS during the posture trial in patients with symptomatic OA deformations. Figure 3 shows that the abduction/adduction ROM decreased for subjects OA2, OA3 and OA4. In contrast, OA1 ab/adduction ROM was already small and physiologically acceptable. Interestingly, the X-ray image for OA3 showed varus deformities. However, the initial angles in the frontal plane suggested that the patient had a valgus knee. The PCA correction seemed to have modified this angle to produce a varus knee, as found in previous X-ray images. In order to statically validate the varus/valgus deformation against its X-ray value, we compared the corrected varus/valgus angle during the posture trial with the x-ray angle, obtained in similar positions. As expected, table 2 shows that the varus/valgus angles after correction were much closer to the x-ray grading than before correction.

**Table 2:** Comparison between the x-ray grading and the varus/valgus angle during the posture trial before (b.c.) and after (a.c.) correction.

	Posture trial angle		X-ray grading
	b.c.	a.c.	
OA 1	4.64 °	4.78 °	5.0 °
OA 2	−8.49 °	−4.33 °	−3.6 °
OA 3	−2.71 °	6.20 °	6.0 °
OA 4	−2.26 °	4.03 °	4.3 °

## Discussion

This study aims to propose a new easy-to-apply method using PCA to correct knee kinematics during gait analysis. Particularly, it seems that PCA provided a better representation of the knee angles, reduced the cross-talk effect and did not alter symptomatic deformations for OA patients. To our knowledge, this study is the first to employ this classical statistical tool in order to improve the accuracy of 3D gait kinematic analysis.

Many studies [11]–[13], [20]–[22] have assessed the influence of errors in aligning the knee flexion/extension axis. All pointed out that misalignment of the CS in the knee joint led to significant errors in abduction/adduction and internal/external rotation angle curves. Several methods for defining the knee joint flexion/extension axis before gait trials have been presented [23]–[27] while other methods have involved post hoc reorientation of the joint CS to correct axis misalignment. Indeed, data may be corrected by rotating the initial CS until abduction/adduction and internal/external rotation are zeroed at the time of maximum knee flexion during gait [29], or between-subject variance may be reduced by minimizing the quadratic variations in the knee abduction/adduction and internal/external rotation angles [28]. This correction, applied to gait data for a small cohort collected using the same protocol that we used [12], [32], was found to appreciably reduce the cross-talk effect. In our study, the corrected joint kinematics gave varus/valgus and axial rotation curves that were similar to those from this method. However, Rivest assumed the flexion/extension angle to be invariant and proposed to minimize ab/adduction and axial rotation angles without reporting these data on the flexion/extension axis [28]. Such a method may delete existing mechanisms of the pathological knee. Since the PCA method uses an objective kinematic criterion for standardization, it is not limited when applied to pathological patients. Besides, the procedure does not have to be implemented during the gait analysis itself and can easily be carried out *a posteriori*, which is useful in the case of large cohorts of patients.

The value of knee varus/valgus ROM of the classical Cardanic angle obtained with the landmark procedure gave data that were out of the range of physiological values for healthy subjects. The errors observed were mainly due to the cross-talk effect, which is assumed to reflect a degree of knee joint flexion-extension axis misalignment [2], [12], [23]. The PCA corrected the kinematics of the joint by reducing the variance in the space defined by the three principal axes. This method was evaluated with respect to three main criteria: (1) the deletion of knee joint angle cross-talk (2) the reduction of variance in the varus/valgus kinematic profile (3) the postural varus/valgus deformation matching the X-ray value for patients with knee osteoarthritis. Indeed, it has been clearly demonstrated that in a stable knee joint, the physiological ROM of knee varus-valgus is small [11], [36]. Furthermore, the kinematic profiles from these studies do not display any obvious evidence of coupling between knee flexion-extension and varus-valgus, unlike that between knee flexion-extension and axial rotation [17], [40]. These findings suggest that the presence of cross-talk, measured as the correlation (r^2^) between knee flexion-extension and varus-valgus kinematic profiles, is not an expected physiological phenomenon but is rather a reflection of knee flexion-extension axis misalignment. Reduction of knee joint cross-talk using correlation (r^2^) and knee varus-valgus variability (δ^2^ab/ad) reduction were therefore considered to be valid criteria to evaluate our correction method [25], [28], [37], [38]. This evaluation was strengthened, in the third criterion, by the comparison between corrected angles and x-ray graded angles of symptomatic valgus/varus deformities for patients with knee OA. Finally, the experimental findings are directly in line with the expected findings as the main goal of PCA is to transform original data of correlated variables into new ones with uncorrelated components. However, such corrections applied to the knee joint should have repercussions on adjacent joint kinematics.

Additionally, it should be noted that knee angles are Euler angles associated with rotational motion between the articulating bones of the knee. Therefore, it might be of interest to completely redefine the knee joint CS with respect to the new knee angle curves determined by the PCA correction. This means modifying both femur and tibia CS dynamically during gait and thereafter recalculating lower body kinematics and kinetics. This would make it possible to analyze the repercussions of this correction on “downstream” results: (i) by calculating new hip and ankle angles, as they derive from the rotation matrix between the knee joint CS (i.e. the femur CS) and the hip or ankle CS (i.e. respectively pelvis and tibia CS), and (ii) by determining the new position of the hip joint center (HJC). More accurate methods have been proposed to locate the HJC [41], [42]. The combination of such methods and PCA correction should be explored in future studies in order to improve the quality of the results in gait analysis.

Furthermore, some of the variability of lower-body kinematics could be attributed to the operator, and specifically to the operator's ability to identify the external anatomical landmarks. Since the current correction removes the errors introduced by the incorrect positioning of markers on anatomical landmarks, one could easily imagine that it could lead to more reproducible data [43]. In this regard, further investigations should be made in order to analyze the impact of this correction on between-session and between-operator variability.

## Conclusion

This paper proposes a post-hoc statistical correction method that is objective, accurate and easy to apply for estimating knee joint kinematics. It is based on the anatomical constraint that the knee articulates principally around one major axis (flexion/extension axis), which allows the use of PCA to retrieve more anatomical knee axes. The findings of this paper, interpreted with an understanding of the knee cross-talk phenomenon, highlight the fact that the correction method produced new rotational axes that correspond more closely to true knee motion.
